# Classification accuracy of blood-based and neurophysiological markers in the differential diagnosis of Alzheimer’s disease and frontotemporal lobar degeneration

**DOI:** 10.1186/s13195-022-01094-5

**Published:** 2022-10-13

**Authors:** Alberto Benussi, Valentina Cantoni, Jasmine Rivolta, Silvana Archetti, Anna Micheli, Nicholas Ashton, Henrik Zetterberg, Kaj Blennow, Barbara Borroni

**Affiliations:** 1 Neurology Unit, Department of Clinical and Experimental Sciences, University of Brescia, P.le Spedali Civili 1, 25123 Brescia, Italy; 2 Neurology Unit, ASST Spedali Civili Brescia, Brescia, Italy; 3 Department of Molecular and Translational Medicine, University of Brescia, Brescia, Italy; 4 Biotechnology Laboratory and Department of Diagnostics, Civic Hospital of Brescia, Brescia, Italy; 5 Casa Di Cura San Francesco, Bergamo, Italy; 6 Institute of Neuroscience and Physiology, Sahlgrenska Academy at University of Gothenburg, Mölndal, Sweden; 7 Wallenberg Centre for Molecular and Translational Medicine, University of Gothenburg, Mölndal, Sweden; 8 King’s College London, Institute of Psychiatry, Psychology & Neuroscience, Maurice Wohl Clinical Neuroscience Institute, London, UK; 9 NIHR Biomedical Research Centre for Mental Health & Biomedical Research Unit for Dementia at South London & Maudsley NHS Foundation, London, UK; 10 Clinical Neurochemistry Laboratory, Sahlgrenska University Hospital, Mölndal, Sweden; 11 UK Dementia Research Institute at UCL, London, UK; 12 Department of Neurodegenerative Disease, UCL Institute of Neurology, London, UK; 13 Hong Kong Center for Neurodegenerative Diseases, Clear Water Bay, Hong Kong, China

**Keywords:** Frontotemporal dementia, Alzheimer’s disease, Serum, Biomarkers, Neurofilament light, GFAP, p-tau, Amyloid, Transcranial magnetic stimulation

## Abstract

**Background:**

In the last decade, non-invasive blood-based and neurophysiological biomarkers have shown great potential for the discrimination of several neurodegenerative disorders. However, in the clinical workup of patients with cognitive impairment, it will be highly unlikely that any biomarker will achieve the highest potential predictive accuracy on its own, owing to the multifactorial nature of Alzheimer’s disease (AD) and frontotemporal lobar degeneration (FTLD).

**Methods:**

In this retrospective study, performed on 202 participants, we analysed plasma neurofilament light (NfL), glial fibrillary acidic protein (GFAP), and tau phosphorylated at amino acid 181 (p-Tau_181_) concentrations, as well as amyloid β42 to 40 ratio (Aβ_1–42_/_1–40_) ratio, using the ultrasensitive single-molecule array (Simoa) technique, and neurophysiological measures obtained by transcranial magnetic stimulation (TMS), including short-interval intracortical inhibition (SICI), intracortical facilitation (ICF), long-interval intracortical inhibition (LICI), and short-latency afferent inhibition (SAI). We assessed the diagnostic accuracy of combinations of both plasma and neurophysiological biomarkers in the differential diagnosis between healthy ageing, AD, and FTLD.

**Results:**

We observed significant differences in plasma NfL, GFAP, and p-Tau_181_ levels between the groups, but not for the Aβ_1–42_/Aβ_1–40_ ratio. For the evaluation of diagnostic accuracy, we adopted a two-step process which reflects the clinical judgement on clinical grounds. In the first step, the best single biomarker to classify “cases” vs “controls” was NfL (AUC 0.94, *p* < 0.001), whilst in the second step, the best single biomarker to classify AD vs FTLD was SAI (AUC 0.96, *p* < 0.001). The combination of multiple biomarkers significantly increased diagnostic accuracy. The best model for classifying “cases” vs “controls” included the predictors p-Tau_181_, GFAP, NfL, SICI, ICF, and SAI, resulting in an AUC of 0.99 (*p* < 0.001). For the second step, classifying AD from FTD, the best model included the combination of Aβ_1–42_/Aβ_1–40_ ratio, p-Tau_181_, SICI, ICF, and SAI, resulting in an AUC of 0.98 (*p* < 0.001).

**Conclusions:**

The combined assessment of plasma and neurophysiological measures may greatly improve the differential diagnosis of AD and FTLD.

## Background

Alzheimer’s disease (AD) and frontotemporal lobar degeneration (FTLD) are a major and increasing global health challenge, with cases estimated to reach 150 million worldwide in 2050 [1], due to the constant increase of elderly people as younger age mortality declines [2]. In this scenario, several priorities are being identified, particularly in view of the development of disease-specific and disease-modifying therapies that target distinct proteinopathies; the foremost concern is to promptly identify patients with a neurodegenerative disorder from healthy ageing with high diagnostic confidence and then discriminate neurodegenerative disorders from one another, to benefit patients with tailored therapies and prognostic counselling.

Currently, validated markers, divided into imaging modalities and cerebrospinal fluid (CSF) measures, are used on clinical grounds and have proven to be highly accurate in diagnosing dementia [3]. However, several drawbacks may limit the use of these markers; thus, they are considered only in selected cases. In particular, some are able to identify AD but are unhelpful in other forms of dementia (i.e. amyloid positron emission tomography [PET] imaging or CSF Aβ_42_ and tau concentrations), and others are not useful in early disease stages at the single subject level (i.e. brain magnetic resonance imaging [MRI]); moreover, the invasiveness of the procedure (i.e. CSF analysis) or the expensiveness (i.e. amyloid PET) may further limit their availability. Notably, the ideal marker, besides having high accuracy and reliability, should be non-invasive, simple to perform, and inexpensive [4].

In the last decade, non-invasive blood-based biomarkers have been extensively studied and refined, showing great potential in the identification of neurodegenerative disorders, even in the prodromal phases of disease [5–9]. Moreover, neurophysiological techniques, such as transcranial magnetic stimulation, have been shown to be non-invasive and accurate in the discrimination of different dementing conditions [10–12].

As each biomarker (blood-based or neurophysiological) has shown great potential for the individualized prediction of neurodegenerative conditions, in the clinical workup of patients with cognitive impairment, it will be highly unlikely that any biomarker will achieve the highest potential predictive accuracy on its own, owing to the multifactorial nature of AD and FTLD and its heterogeneous clinical presentations. Consequently, there is a necessity to identify a combination of measures that should be integrated to produce the most accurate, easily accessible, non-invasive, and cost-effective diagnostic algorithm for the classification of common neurodegenerative disorders.

The aim of the current study was to examine the diagnostic accuracy of blood-based and neurophysiological biomarkers, both taken individually and in combination with other biomarkers, in a two-step phase. In the first step, patients with neurodegenerative dementia, i.e. AD or FTLD, should be discriminated from healthy controls, whilst in the second step, AD should be identified from other conditions such as FTLD.

## Materials and methods

### Participants

This retrospective study included 202 participants from the Centre for Neurodegenerative Disorders, Department of Clinical and Experimental Sciences, University of Brescia, Brescia, Italy.

The cohort consisted of 127 patients meeting probable clinical criteria for a syndrome in the FTLD spectrum, namely 67 behavioural variant frontotemporal dementia (bvFTD), 44 primary progressive aphasia (PPA), 7 corticobasal syndrome (CBS) and 9 progressive supranuclear palsy (PSP) [13–16]. Moreover, 48 patients fulfilling clinical criteria for AD [17] and 27 healthy controls (HC), recruited among spouses or caregivers, were included as well.

Each patient underwent a neurological evaluation, routine laboratory examination, and a neuropsychological and behavioural assessment. In all cases, the diagnosis was supported by brain structural imaging, whilst CSF concentrations of tau, p-Tau_181_, and Aβ_1–42_ were measured in a subset of cases (*n* = 142, 64.5%), as previously reported [18]. Furthermore, in familial cases (based on the presence of at least one dementia case among the first-degree relatives) and early-onset sporadic cases, genetic screening for *GRN*, *C9orf72*, and *MAPT* P301L mutations was performed; given the low frequency of *MAPT* mutations in Italy [19], we considered only the P301L mutation, and we sequenced the entire *MAPT* gene only in selected cases.

Each participant underwent blood collection for measurements of serum NfL, GFAP, p-Tau_181_, Aβ_1–42_, and Aβ_1–40_ biomarkers. Moreover, each included patient underwent transcranial magnetic stimulation (TMS) protocols that partially and indirectly reflect the activity of several neurotransmitters, including GABA_A_ by short-interval intracortical inhibition (SICI), glutamate by intracortical facilitation (ICF), GABA_B_ by long-interval intracortical inhibition (LICI), and acetylcholine by short-latency afferent inhibition (SAI) [20–22].

### Clinical evaluation

At baseline, patients underwent a standardized neuropsychological battery which included the Mini-Mental State Examination (MMSE), the short story recall test, the Rey complex figure (copy and recall), the phonemic and semantic fluencies, the token test, the clock-drawing test, and the trail-making test (part A and part B) [23]. Disease severity was assessed with the clinical dementia rating plus National Alzheimer’s Coordinating Center (NACC) behaviour and language domains (CDR plus NACC FTLD) global and sum of boxes, whilst the level of functional independence was assessed with the basic activities of daily living (BADL) and the instrumental activities of daily living (IADL) questionnaire. Furthermore, neuropsychiatric and behavioural disturbances were evaluated with the neuropsychiatric inventory (NPI) [24].

HCs underwent a brief standardized neuropsychological assessment (MMSE ≥ 27/30); psychiatric or other neurological illnesses were considered exclusion criteria.

### Serum biomarkers

Plasma was collected by venipuncture, processed, and stored in aliquots at − 80 °C according to the standardised procedures. Plasma NfL concentration was measured using a commercial single-molecule array (Simoa) NF-Light® assay (Quanterix, Billerica, MA) according to the manufacturer’s instructions [25]. Plasma p-Tau_181_ concentration was measured using an in-house Simoa assay developed at the University of Gothenburg [8]. In brief, the capture antibody (AT270, Invitrogen) which is specific for the threonine-181 phosphorylation site [26] was coupled to paramagnetic beads whilst the detector antibody (Tau12, BioLegend) was raised against the N-terminal epitope amino acid 6-QEFEVMEDHAGT-18 on human tau protein. Detailed analytical procedures and assay validation have been previously described [8]. Plasma GFAP, Aβ_1–42_, and Aβ_1–40_ concentrations were measured using commercial Simoa assays (Quanterix, Billerica, MA). All measurements were carried out using an HD-X analyser (Quanterix, Boston, MA) in one round of experiments, using one batch of reagents with operators blinded to clinical information.

### Transcranial magnetic stimulation

A TMS figure-of-eight coil (each loop diameter 70 mm – D70^2^ coil) connected to a monophasic Magstim Bistim^2^ system (Magstim Company, Oxford, UK) was employed for all TMS paradigms, as previously reported [27]. Electromyographic (EMG) recordings were performed from the first dorsal interosseous muscle using 9 mm diameter, Ag–AgCl surface-cup electrodes. The active electrode was placed over the muscle belly and the reference electrode over the metacarpophalangeal joint of the index finger. Responses were amplified and filtered at 20 Hz and 2 kHz with a sampling rate of 5 kHz.

Resting motor threshold (RMT) was determined on the left motor cortex as the minimum intensity of the stimulator required to elicit motor evoked potentials (MEPs) with a 50-μV amplitude in 50% of 10 consecutive trails, recorded during full muscle relaxation.

SICI-ICF, LICI, and SAI were studied using a paired-pulse technique, employing a conditioning test design. For all paradigms, the test stimulus (TS) was adjusted to evoke a MEP of approximately 1-mV amplitude.

For SICI and ICF, the conditioning stimulus (CS) was adjusted at 70% of the RMT, employing multiple interstimulus intervals (ISIs), including 1, 2, and 3 ms for SICI and 7, 10, and 15 ms for ICF [28, 29]. LICI was investigated by implementing two supra-threshold stimuli, with the CS adjusted at 130% of the RMT, employing ISIs of 50, 100, and 150 ms [30]. SAI was evaluated employing a CS of single pulses (200 μs) of electrical stimulation delivered to the right median nerve at the wrist, using a bipolar electrode with the cathode positioned proximally, at an intensity sufficient to evoke a visible twitch of the thenar muscles [31]. Different ISIs were implemented (0, + 4), which were fixed relative to the N20 component latency of the somatosensory evoked potential of the median nerve.

For each ISI and for each protocol, ten different paired CS-TS stimuli and fourteen control TS stimuli were delivered to all participants in a pseudo-randomized sequence, with an inter-trial interval of 5 secs (± 10%).

The conditioned MEP amplitude, evoked after delivering a paired CS-TS stimulus, was expressed as a percentage of the average control MEP amplitude. The average values for SICI (1, 2, 3 ms ISI), ICF (7, 10, 15 ms ISI), LICI (50, 100, 150 ms ISI), and SAI (0, + 4 ms ISI) were used for analysis.

Stimulation protocols were conducted in a randomized order. Audio-visual feedback was provided to ensure muscle relaxation during the entire experiment, and trials were discarded if EMG activity exceeded 100 μV in the 250 ms prior to TMS stimulus delivery. Less than 5% of trials were discarded for each protocol. All of the participants were capable of following instructions and reaching complete muscle relaxation; if, however, the data was corrupted by patient movement, the protocol was restarted, and the initial recording was rejected.

### Statistical analysis

Continuous and categorical variables are reported as median (interquartile range) and *n* (%), respectively. Differences in clinical variables, biomarker concentrations, and neurophysiological measures between the groups were compared by Kruskal–Wallis *H* test, Mann–Whitney *U* test, *χ*^2^ test, or Fisher’s exact test where appropriate.

A rank-based partial correlation was run to assess the relationship between serum biomarkers, clinical variables, and TMS measures, correcting for age.

To identify the most accurate combination of biomarkers, the initial model selection was performed using the R package MuMIn, which tests all different combinations of variables and then ranks the models according to the Akaike Information Criterion (AIC). AIC is a model performance metric which considers the trade-off between model fit and sparsity. Whilst *R*^2^ and AUC explain how well a model performs on the observed data, AIC rather explains how well a model would perform on unseen data. In other words, the larger the difference in AIC values between the two models, the less likely it is that the model with the higher AIC value would provide better predictive performance on unseen data than the model with the lower AIC value. The model with the lowest AIC was selected as the model with the best trade-off between fit and complexity and then a stepwise removal of variables was performed as long as the ΔAIC was < 2 from the model with the best fit to end up with a “parsimonious model” [32, 33]. Further variables were removed using a stepwise procedure in subsequent models to illustrate the added value of different variables and combinations of variable. Receiver operating characteristics (ROC) curve analyses were plotted, and the area under the curve (AUC) including 95% confidence interval (CI) values are reported. Comparisons of AUC were performed using DeLong statistics.

A two-sided *p*-value < 0.05 was considered significant and corrected for multiple comparisons using false discovery rate (FDR) when appropriate. Statistical analyses were performed using IBM SPSS (v.25.0.2), GraphPad Prism (v.9.3.1), and R (v.4.21).

### Data availability

All study data, including raw and analysed data, and materials will be available from the corresponding author, B.B., upon reasonable request.

## Results

### Participant characteristics

**Fig. 1:**
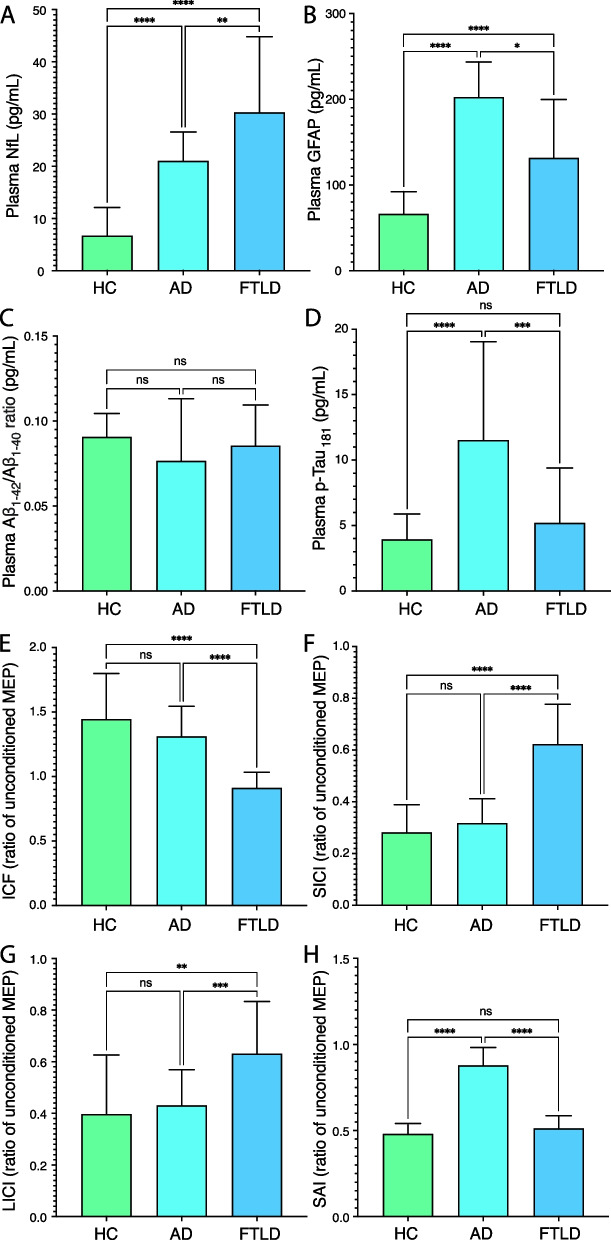
Serum biomarker concentrations and neurophysiological measures in participants by clinical diagnosis. **A** PLasma NfL. **B** GFAP. **C** Aβ_1–42_/Aβ_1–40_ ratio. **D** p-Tau_181_. **E** average SICI. **F** ICF. **G** LICI. **H** SAI values in participants by clinical diagnosis. HC, healthy controls; AD, Alzheimer’s disease; FTLD, frontotemporal lobar degeneration; NfL, neurofilament light; GFAP, glial fibrillary acidic protein; SICI, average short-interval intracortical inhibition (1, 2, 3 ms); ICF, average intracortical facilitation (7, 10, 15 ms); LICI, average long-interval intracortical inhibition (50, 100, 150 ms); SAI, average short-latency afferent inhibition (0, + 4 ms) expressed as the ratio of the unconditioned motor evoked potential (MEP). Bar graphs represent the median values, and error bars represent the interquartile range. *p < 0.050; **p < 0.010; ***p < 0.001 after multiple-comparisons corrected post hoc tests

A total of 202 patients were included in the present study, namely 127 FTLD, 48 AD patients, and 27 HC. Demographic and clinical characteristics, fluid biomarker levels, and neurophysiological measures are reported in Table 1. We observed significant differences in NfL, GFAP, and p-Tau_181_ levels between the groups but not for the Aβ_1–42_/Aβ_1–40_ ratio. NfL and GFAP levels were significantly increased in FTLD and AD, compared to HC (*p* = 0.005 and *p* < 0.001, respectively), whilst p-Tau_181_ levels were significantly increased in AD patients compared to FTLD and HC (both *p* < 0.001) (see Fig. 1). Regarding neurophysiological measures, AD showed decreased SAI compared to FTLD and HC (both *p* < 0.001), whilst FTLD showed reduced SICI, ICF, and LICI compared to AD and HC (both *p* < 0.001) (see Fig. 1).

**Table 1:** Demographic and clinical characteristics of HC, AD, and FTLD patients

Variables	HC	AD	FTLD	*p*-value
Number	27	48	127	
Age, years	48.0 (38.0–68.0)^a, b^	68.5 (61.8–73.0)^c^	64.0 (58.0–70.0)^c^	< 0.001
Sex, female %	55.6	44.3	47.8	n.s
Education, years	11.0 (8.0–13.0)	8.0 (7.5–13.0)	10.0 (8.0–13.0)	n.s
Age at onset, years	–	65.5 (59.0–71.0)	62.0 (55.0–68.0)	n.s
Disease duration, years	–	2.0 (1.0–3.0)	2.0 (1.0–3.0)	n.s
MMSE	30.0 (29.0–30.0)	24.5 (22.0–27.0)	26.0 (21.0–27.0)	< 0.001
NPI	–	5.0 (2.0–8.0)^b^	11.0 (8.0–18.0)^a^	< 0.001
CDR plus NACC FTLD	–	2.0 (1.5–3.5)^b^	4.0 (2.0–8.0)^a^	< 0.001
BADL (lost)	0.0 (0.0–0.0)^b^	0.0 (0.0–0.0)^b^	0.0 (0.0–1.0)^a, c^	0.001
IADL (lost)	0.0 (0.0–0.0)^a, b^	0.0 (0.0–1.0)^c^	1.0 (0.0–2.0)^c^	< 0.001
Serum biomarkers				
NfL (pg/mL)	6.7 (4.9–12.1)^a, b^	21.1 (14.1–26.6)^b, c^	30.3 (19.2–44.8)^a, c^	< 0.001
GFAP (pg/mL)	66.3 (40.7–92.2)^a, b^	202.5 (130.2–243.5)^b, c^	131.8 (94.6–199.6)^a, c^	< 0.001
p-Tau_181_ (pg/mL)	4.0 (2.6–5.9)^a^	11.5 (6.5–19.0)^b, c^	5.2 (3.2–9.4)^a^	< 0.001
Aβ_1–42_/Aβ_1–40_ ratio	0.09 (0.08–0.10)	0.08 (0.07–0.11)	0.09 (0.07–0.11)	n.s
TMS measures				
SICI	0.28 (0.16–0.39)^b^	0.32 (0.25–0.41)^b^	0.62 (0.53–0.78)^a, c^	< 0.001
ICF	1.45 (1.33–1.80)^b^	1.31 (1.18–1.54)^b^	0.91 (0.77–1.03)^a, c^	< 0.001
LICI	0.40 (0.23–0.63)^b^	0.43 (0.25–0.57)^b^	0.63 (0.43–0.83)^a, c^	< 0.001
SAI	0.48 (0.40–0.54)^a^	0.88 (0.78–0.98)^b, c^	0.51 (0.47–0.59)^a^	< 0.001

Results are expressed as median (interquartile range), unless otherwise specified

*FTLD* Frontotemporal lobar degeneration, *AD* Alzheimer’s disease, *HC* Healthy controls, *MMSE* Mini-Mental State Examination, *NPI* Neuropsychiatric inventory, *CDR plus NACC FLTD* Clinical dementia rating plus National Alzheimer’s Coordinating Center behaviour and language domains, *BADL* Basic activities of daily living, *IADL* Instrumental activities of daily living, *NfL* Neurofilament light chain, *GFAP* Glial fibrillary acidic protein, *Aβ* Amyloid beta, *TMS* Transcranial magnetic stimulation, *SICI* Average short-interval intracortical inhibition (1, 2, 3 ms), *ICF* Average intracortical facilitation (7, 10, 15 ms), *LICI* Average long-interval intracortical inhibition (50, 100, 150 ms), *SAI* Average short-latency afferent inhibition (0, + 4 ms) expressed as the ratio of the unconditioned motor evoked potential

^a^Significant difference compared to AD

^b^Significant difference compared to FTD

^c^Significant difference compared to HC

### Correlations between serum biomarkers, neuropsychological scores, and neurophysiological measures

Age-corrected biomarker concentrations correlated with several neuropsychological scores. In particular, we observed significant correlations between NfL and CDR plus NACC FTLD sum of boxes (*r* = 0.37, *p* < 0.001), NPI (*r* = 0.28, *p* < 0.001), and MMSE scores (*r* =  − 0.19, *p* = 0.013), but also with neurophysiological scores as SICI (*r* = 0.17, *p* = 0.025) and SAI (*r* =  − 0.21, *p* = 0.006). GFAP also showed significant correlations with MMSE scores (*r* =  − 0.34, *p* < 0.001) and LICI (*r* =  − 0.16, *p* = 0.036).

p-Tau_181_ values strongly correlated with the Aβ_1–42_/Aβ_1–40_ ratio (*r* =  − 0.30, *p* < 0.001), whilst NfL strongly correlated with GFAP levels (*r* = 0.29, *p* < 0.001).

### Diagnostic accuracy of blood-based biomarkers and neurophysiological measures

For the evaluation of diagnostic accuracy, we adopted a two-step process which reflects a clinical judgement on clinical grounds. The first step allows us to classify each subject as “case” (i.e. patient with dementia) or “control”. If the subject falls into the “case” category, the next order of classification was considered, and the AD vs FTLD classifier was carried out.

**Fig. 2:**
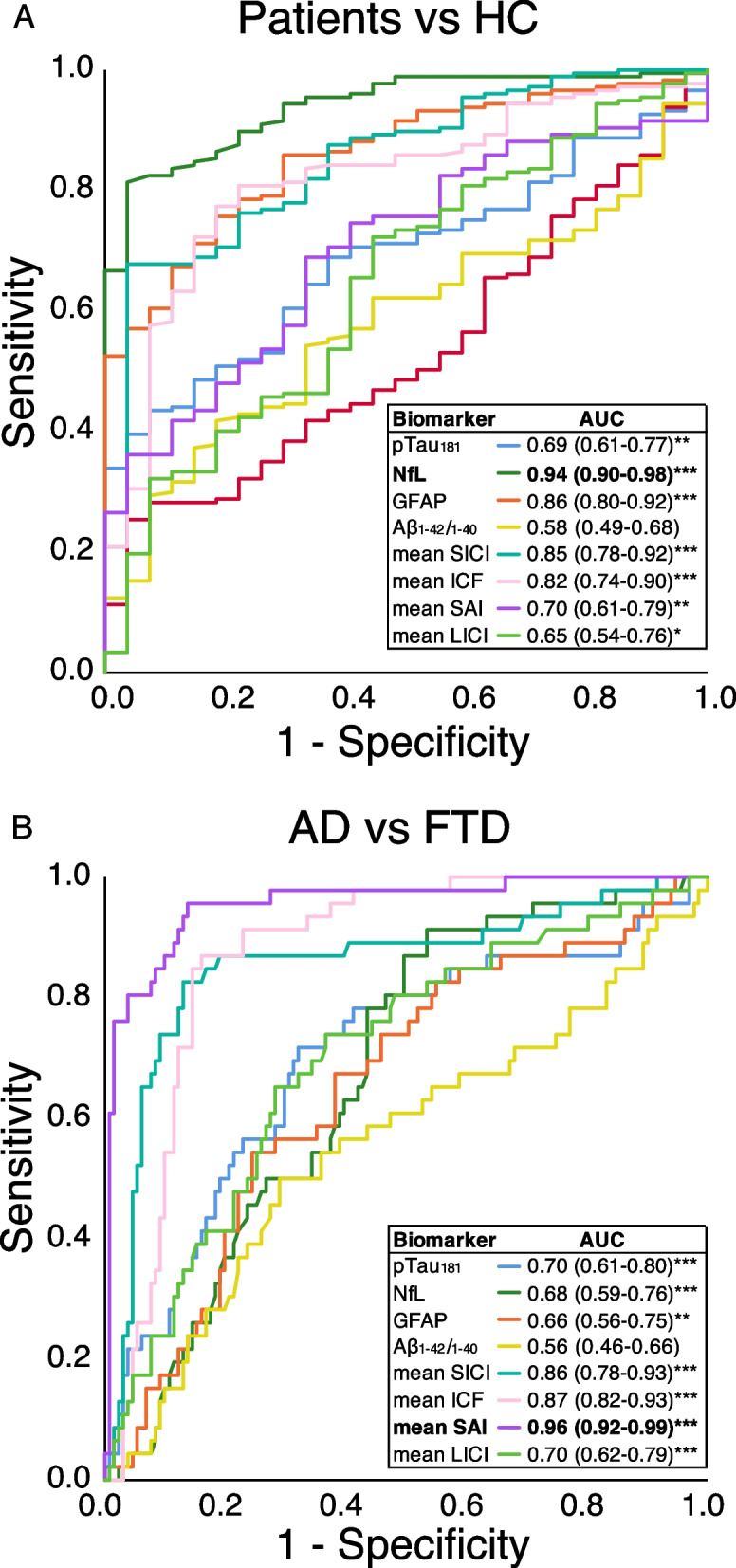
ROC curve analysis. ROC curves for differentiating **A** “cases” vs “controls” and **B** AD vs FTLD patients. ROC, receiver operating characteristic; AUC, area under the curve; HC, healthy controls; AD, Alzheimer’s disease; FTLD, frontotemporal lobar degeneration; NfL, neurofilament light; GFAP, glial fibrillary acidic protein; SICI, average short-interval intracortical inhibition (1, 2, 3 ms); ICF, average intracortical facilitation (7, 10, 15 ms); LICI, average long-interval intracortical inhibition (50, 100, 150 ms); SAI, average short-latency afferent inhibition (0, + 4 ms) expressed as the ratio of the unconditioned motor evoked potential (MEP)

As shown in Fig. 2A, the best single biomarker to classify “cases” vs “controls” was NfL (AUC 0.94 [95% CI 0.90–0.98], *p* < 0.001), followed by GFAP (AUC 0.86 [95% CI 0.80–0.92], *p* < 0.001). In the second step, as shown in Fig. 2B, the best single biomarker to classify AD vs FTLD was SAI (AUC 0.96 [95% CI 0.92–0.99], *p* < 0.001), followed by ICF (AUC 0.87 [95% CI 0.82–0.93], *p* < 0.001).

We subsequently applied a data-driven model selection process to choose the best combination of biomarkers with the lowest AIC, removing as many variables as possible whilst maintaining a similar model performance defined as being within two AIC points of the lowest AIC model identified (ΔAIC < 2). Thereafter, variables were removed further in a stepwise procedure to examine the performance of the more basic models (i.e. the most accurate model with exclusively neurophysiological or blood-based biomarkers, a model with only a single neurophysiological or blood-based biomarker, and a model with the combination of one neurophysiological and one blood-based biomarker).

**Fig. 3:**
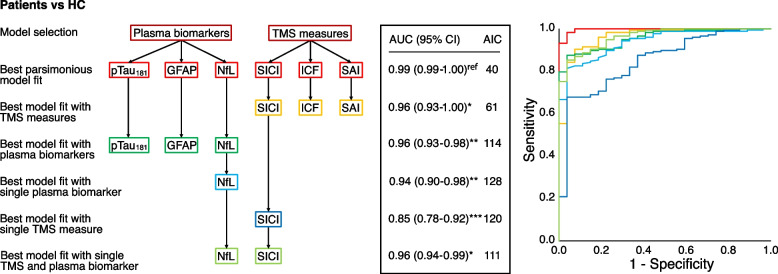
Model selection process and performance of predicting “cases” vs “controls”. The logistic regression model selection process with the best model fit by data-driven selection with the lowest AIC. The parsimonious model shows the model that had a similar performance (ΔAIC < 2) with as few significant predictors as possible. In subsequent models, predictors were removed in a stepwise procedure. Comparisons between AUCs were performed using DeLong statistics, *p < 0.05, **p < 0.010, ***p < 0.001 compared to the best model fit. ROC curves combining different models are plotted on the right side of the figure. AUC, area under the curve; AIC, Akaike Information Criterion; HC, healthy controls; NfL, neurofilament light; GFAP, glial fibrillary acidic protein; SICI, average short-interval intracortical inhibition (1, 2, 3 ms); ICF, average intracortical facilitation (7, 10, 15 ms); SAI, average short-latency afferent inhibition (0, + 4 ms) expressed as the ratio of the unconditioned motor evoked potential (MEP)

The best model for classifying “cases” vs “controls” included the predictors p-Tau_181_, GFAP, NfL, SICI, ICF, and SAI, resulting in an AUC of 0.99 (95% CI 0.99–1.00), *p* < 0.001 (see Fig. 3). By selecting exclusively blood-based biomarkers (p-Tau_181_, GFAP, and NfL) or neurophysiological measures (SICI, ICF, and SAI), we obtained very similar results (AUC 0.96 [95% CI 0.93–0.98], *p* < 0.001 and AUC 0.96 [95% CI 0.93–1.00], *p* < 0.001, respectively). Even higher accuracy was reached by combining the best blood-based biomarker (NfL) (AUC 0.94 [95% CI 0.90–0.98], *p* < 0.001) with the best neurophysiological measure (SICI) (AUC 0.85 [95% CI 0.78–0.92], *p* < 0.001), resulting in an AUC of 0.96 (95% CI 0.94–0.99), *p* < 0.001 (see Fig. 3).

**Fig. 4:**
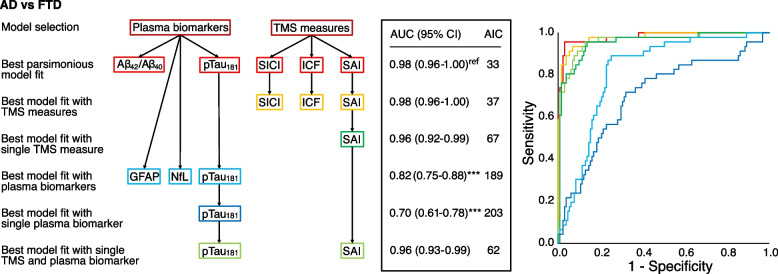
Model selection process and performance of predicting AD vs FTLD. The logistic regression model selection process with the best model fit by data-driven selection with the lowest AIC. The parsimonious model shows the model that had a similar performance (ΔAIC < 2) with as few significant predictors as possible. In subsequent models, predictors were removed in a stepwise procedure. Comparisons between AUCs were performed using DeLong statistics, *p < 0.05, **p < 0.010, ***p < 0.001 compared to the best model fit (ref). ROC curves combining different models are plotted on the right side of the figure. AUC, area under the curve; AIC, Akaike Information Criterion; AD, Alzheimer’s disease; FTLD, frontotemporal lobar degeneration; NfL, neurofilament light; GFAP, glial fibrillary acidic protein; SICI, average short-interval intracortical inhibition (1, 2, 3 ms); ICF, average intracortical facilitation (7, 10, 15 ms); SAI, average short-latency afferent inhibition (0, + 4 ms) expressed as the ratio of the unconditioned motor evoked potential (MEP)

For the second step, classifying AD from FTD, the best model included the combination of Aβ_1–42_/Aβ_1–40_ ratio, p-Tau_181_, SICI, ICF, and SAI, resulting in an AUC of 0.98 (95% CI 0.96–1.00), *p* < 0.001 (see Fig. 4). Considering exclusively blood-based biomarkers, the best model included GFAP, p-Tau_181_, and NfL (AUC 0.82 [95% CI 0.75–0.88], *p* < 0.001), whilst the best model with exclusively neurophysiological measures included SICI, ICF, and SAI (AUC 0.98 [95% CI 0.96–1.00], *p* < 0.001). By combining the best blood-based biomarker (p-Tau_181_, AUC 0.70 [95% CI 0.61–0.78], *p* < 0.001) with the best neurophysiological measure (SAI, AUC 0.96 [95% CI 0.93–0.99], *p* < 0.001), we obtained an AUC of 0.96 (95% CI 0.93–0.99), *p* < 0.001 (see Fig. 4).

## Discussion

In the last years, a giant step forward has been made in identifying new diagnostic markers in neurodegenerative dementias, ranging from imaging and neurophysiology to biological measures. However, to spread the use of these markers extensively, beyond research referral centres, they need to be easily accessible, inexpensive, not time-consuming, and procedurally easy to perform.

In this view, the recently proposed blood-based biomarkers hold the potential to be available in any centre [34], and TMS intracortical excitability measures may be considered an adjunctive screening tool to be performed during outpatient visits [35].

In the present work, we aimed at assessing the classification accuracy of these markers, taken individually or in combination, in a large sample of consecutive patients with AD or FTLD and healthy controls. We adopted an intuitive and straightforward step-by-step approach resembling clinical reasoning, and at first, we aimed at discriminating ongoing neurodegenerative dementia from healthy ageing, and then we sought to identify AD cases from other conditions such as FTLD. We considered the best parsimonious model fit, the best model fit with either blood-based or TMS markers, and the best model fit with a single marker.

Our results supported the usefulness of blood NfL dosage as the first screening tool to classify patients with neurodegenerative dementia as compared to healthy subjects (AUC = 0.94), with a further slight increase in accuracy when it was combined with other blood-based biomarkers, such as GFAP or pTau_181_ (AUC = 0.96) or with TMS assessment indirectly evaluating GABAergic neurotransmission, i.e. SICI (AUC = 0.96) (see Figs. 2 and 3). On the other hand, in this cohort, TMS intracortical excitability measures indirectly assessing cholinergic dysfunction, i.e. SAI, were found to be the best single marker to classify AD compared to FTLD patients (AUC = 0.96). In this case, minor further improvements in classification accuracy were obtained when considering all TMS measures, i.e. SICI and ICF (AUC = 0.98) or Ab_1–42_/Ab_1–40_ ratio and pTau_181_ markers (AUC = 0.98) (see Figs. 2 and 4).

Taken together, these findings confirm and extend previous data, corroborating the utility of non-invasive and cost-effective markers on clinical grounds in diagnosing patients with dementia and dementia subtypes, and suggest a specific use of these tools depending on the clinical question. Compared to well-established diagnostic markers, such as CSF or PET tracers [36], this approach had a robust performance and similar accuracy, when markers were considered individually and even more when assessed in combination.

There has been great progress in validating blood-based biomarkers for individualized prediction of neurodegenerative diseases, and it has been widely and consistently demonstrated that serum/plasma NfL and GFAP are markers of neurodegeneration and astrogliosis, respectively [36, 37], even though not sufficiently helpful in discriminating between AD and FTLD [38, 39]. Conversely, plasma Aβ_1–42_/Aβ_1–40_ ratio or pTau_181_ are able to identify AD with high accuracy as compared to other neurodegenerative disorders [6, 40]. We indeed reported a twofold increase of plasma pTau_181_ in AD as compared to FTLD and HC, but we failed to demonstrate significant differences in Ab42/Ab40 ratio between the groups (see Table 1 or Fig. 1), possibly due to the methodological differences in sampling procedures [40, 41] or insufficient robustness (too low fold change between cases with and without brain amyloidosis) of this biomarker [42].

Along with blood-based markers, TMS intracortical connectivity measures, which rely on the biological bases of diseases and their associated specific neurotransmitter impairment, are able to identify a now well-established cholinergic deficit in AD and a significant impairment in GABA and glutamatergic circuits in FTLD [43–46]. Indeed, SAI, a marker of sensorimotor integration, has been shown to partially reflect the activity of cholinergic circuits [47]. Furthermore, SICI is considered to reflect short-lasting postsynaptic inhibition mediated through the GABA_A_ receptors at the level of local interneurons, whilst ICF is thought to represent a net facilitation most likely mediated by glutamatergic NMDA receptors [20, 28, 31].

## Limitations

We acknowledge that this study entails some limitations. First, the generalizability of these findings needs to be further demonstrated in future studies. Second, other recently proposed blood biomarkers may be considered in the near future, and the model may be further refined also considering different assays on the market. Third, we did not have neuropathological confirmation of the present case series; however, each subject underwent comprehensive clinical and neuropsychological evaluation along with structural and functional imaging assessment. Finally, we did not compare the performance of blood-based and neurophysiological markers with more validated determinations in CSF or with imaging measures.

## Conclusions

Despite these limitations, the implementation of this model may be proposed as the first screening tool in subjects with the suspected cognitive decline with cost benefits, especially in primary care centres. These findings support the use of blood-based biomarkers and TMS intracortical excitability measures to identify patients who may undergo secondary CSF or imaging testing. Future studies should evaluate their performances in the prodromal stages of dementia.

## Data Availability

All study data, including raw and analysed data, and materials will be available from the corresponding author, B.B., upon reasonable request.
